# Watching ultrafast responses of structure and magnetism in condensed matter with momentum-resolved probes

**DOI:** 10.1063/1.4996176

**Published:** 2017-12-22

**Authors:** S. L. Johnson, M. Savoini, P. Beaud, G. Ingold, U. Staub, F. Carbone, L. Castiglioni, M. Hengsberger, J. Osterwalder

**Affiliations:** 1 Institute for Quantum Electronics, Eidgenössische Technische Hochschule (ETH) Zürich, CH-8093 Zurich, Switzerland; 2 Paul Scherrer Institut, CH-5032 Villigen, Switzerland; 3 Laboratory for Ultrafast Microscopy and Electron Scattering, ICMP, Ecole Polytechnique Fédérale de Lausanne (EPFL), CH-1015 Lausanne, Switzerland; 4 Department of Physics, University of Zurich, CH-8057 Zurich, Switzerland

## Abstract

We present a non-comprehensive review of some representative experimental studies in crystalline condensed matter systems where the effects of intense ultrashort light pulses are probed using x-ray diffraction and photoelectron spectroscopy. On an ultrafast (sub-picosecond) time scale, conventional concepts derived from the assumption of thermodynamic equilibrium must often be modified in order to adequately describe the time-dependent changes in material properties. There are several commonly adopted approaches to this modification, appropriate in different experimental circumstances. One approach is to treat the material as a collection of quasi-thermal subsystems in thermal contact with each other in the so-called “N-temperature” models. On the other extreme, one can also treat the time-dependent changes as fully coherent dynamics of a sometimes complex network of excitations. Here, we present examples of experiments that fall into each of these categories, as well as experiments that partake of both models. We conclude with a discussion of the limitations and future potential of these concepts.

## I. INTRODUCTION

The idea of using short, intense pulses of light to transiently modify solid-state materials offers unique opportunities to understand the interactions which underlie many of the key properties that are of interest in modern materials science. This is valuable not only for a better fundamental physical understanding, but the strongly non-equilibrium pathways explored are potentially useful for technological purposes such as data storage and signal processing in the solid state.

These twin goals of both fostering a better understanding of fundamental material interactions and exploring the possibility of using light pulses to control material properties in a useful way have driven a considerable amount of recent experimental research in optically induced dynamics in solid state materials. Here, we review one specific aspect of this recent work, where changes are stimulated by strong electromagnetic interactions and characterized by methods employing momentum-resolved spectroscopy with x-rays and photoelectrons to directly and unambiguously extract information on the long-range order of atomic structure and magnetism.

In equilibrium, the long-range order of condensed matter is often considered to be a function of thermodynamic state variables. A typical example of this is the phenomenological Landau theory for second-order phase transitions.^1^ Under Landau theory, changes in order associated with a second-order transition are understood in terms of the minimization of an appropriate free energy with respect to an order parameter. For illustrative purposes, we can take the example of a ferromagnetic-paramagnetic phase transition at a constant volume. Here, the appropriate free energy is the Helmholtz free energy *F* and the order parameter is the magnetization density *m*. For a given fixed temperature, Landau theory assumes that *F* can be written as a series expansion in even orders of *m*. Accepting terms to fourth order, $F\approx F_{0}+\alpha m^{2}+\frac{1}{2}\beta m^{4}$, where the coefficients *F*_0_, *α*, and *β* are functions of the temperature *T*. For α>0 and β>0, there is only one minimum of *F* at *m* = 0, corresponding to the high-temperature paramagnetic phase. For α<0 and β>0, there are two equivalent minima of *F* at $m=\pm \sqrt{\alpha /\beta }$, corresponding to the low-temperature ferromagnetic (FM) phase. The phase transition is understood in terms of a zero-crossing of *α* as the temperature increases through a transition temperature *T_c_*. Assuming that α(T) is analytic, we can write $\alpha (T)=\alpha_{0}(T-T_{c})$ where *α*_0_ is a positive constant.

While this and similar kinds of treatments work in many cases to describe order changes under conditions of thermodynamic equilibrium, they generally fail conceptually to describe changes in the order of a system when driven strongly out of thermodynamic equilibrium, where state variables such as temperature and entropy are not well defined. Historically, there have been several different approaches to understanding time-dependent order in such systems. One approach is to divide the material into different “subsystems,” each of which are assumed to be separately in thermodynamic equilibrium at all relevant times. It is then assumed that each of these subsystems is in some kind of thermal contact and exchange energy, ultimately reaching a thermodynamic equilibrium for the entire system. Another approach, appropriate for quite different situations, is to eschew statistical methods entirely and try to treat the system and its orders in terms of the coherent, classical-like evolution of a system in a suddenly modified potential energy landscape. A nearly trivial example of this would be describing the motion of a classical pendulum where the pivot point has been suddenly displaced over a time scale short compared with the period of the pendulum. Obviously, not all out-of-equilibrium situations lend themselves to a complete description using one of these approaches, resulting in numerous hybrid approaches where part of the material is treated as a statistical subsystem and other degrees of freedom are treated as coherent dynamics. For the purposes of this review, we are concerned with systems that have been brought out of equilibrium by some kind of interaction with an intense pulse of light with a very short duration, ranging from 50 fs to 2 ps. The kind of conceptual framework that is appropriate to describe the behavior of the system depends strongly on the mechanism of the interaction of the light pulse with the material, as well as on the properties of the material itself.

Rather than attempt to construct a comprehensive review of activity in this area, we instead focus on some representative examples related to our own recent work that illustrate the conceptual frameworks that are currently applied to understand out-of-equilibrium time dependence in condensed matter systems. We categorize these studies based on the degree to which the time-dependence is described by the evolution of statistical, quasi-thermal distributions versus coherent dynamics that correspond to the solution of classical-like equations of motion. We first discuss experimental studies where the time evolution of the system is treated in the framework of interacting thermal subsystems. We then proceed to review several examples where the time-dependence is understood in a hybrid framework, where part of the system is treated statistically and other parts are treated deterministically. Finally, we touch on some examples where the complete time-dependence is considered as a coherent, deterministic process. We will at the end conclude with a discussion of how we see the future developments in both understanding and controlling changes in long range order for solid-state systems using light.

## II. INCOHERENT TIME-DEPENDENCE: STATISTICAL SUBSYSTEMS IN THERMAL CONTACT

**FIG. 1.**
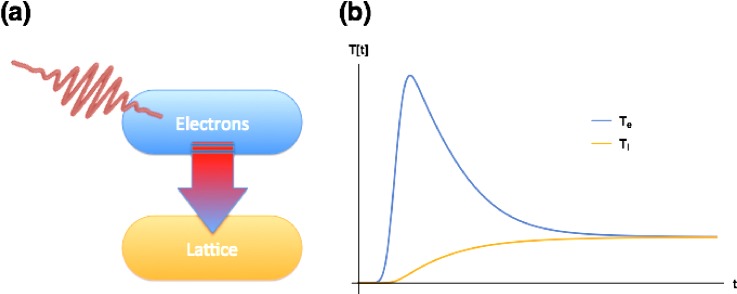
The two-temperature model of electron–lattice interaction, as applied to metallic systems. (a) Conceptual sketch of the model. The laser acts as a heat source for the electron subsystem, which is assumed to thermalize instantaneously to a strongly elevated temperature. This electronic subsystem is in thermal contact with the lattice subsystem, and as time progresses, there is heat exchange as the two subsystems equilibrate. (b) One possible time-dependence for the electronic and lattice temperatures *T_e_* and *T_l_*, where the heat capacity of the lattice $c_{v,l}$ is constant and much larger than the electronic heat capacity $c_{v,e}$. The decay of *T_e_* and the associated rise of the *T_l_* is determined by the coupling strength between the two subsystems.

We begin with a brief overview of common statistical models of time-dependence in out-of-equilibrium materials where ultrashort laser pulses have heated at least part of the electronic subsystem strongly. The first application of such a model to the problem of laser-heated materials is credited to Anisimov, Kapeliovich, and Per el'man who applied a two-temperature model of interacting electron and lattice subsystems to describe transient thermionic emission from metals excited with picosecond laser pulses.^2^ The basic concept of the model is illustrated in Fig. 1. The ultrafast laser is assumed to interact only with the electronic subsystem, acting as a strong but brief heat source. This leads to a dramatic increase in the electronic temperature, given by the relation

$$Q=\int_{T_{0}}^{T_{e}^{\prime }}c_{v,e}(T)dT,$$ (1)

where *Q* is the heat deposited by the laser pulse, $c_{v,e}(T)$ is the temperature-dependent electronic specific heat, *T*_0_ is the initial temperature of the system, and $T_{e}^{\prime }$ is the electronic temperature just after the pulse. Immediately after the pulse, we then have a very high temperature electronic subsystem in thermal contact with a lattice subsystem that is still at the initial temperature of the system as a whole. Since the two subsystems are in thermal contact, heat will flow to the lattice subsystem at a rate

$$Q_{e,l}=\gamma (T_{e}-T_{l}),$$ (2)

where *γ* is a coupling constant, and *T_e_* and *T_l_* are the instantaneous temperatures of the electron and lattice, respectively. Since the process as a whole is adiabatic, this heat transfer in turn will act to increase *T_l_* and decrease *T_e_* according to the coupled equations

$$c_{v,e}(T)\frac{dT_{e}}{dt}=-\gamma (T_{e}-T_{l}),$$ (3)

$$c_{v,l}(T)\frac{dT_{l}}{dt}=\gamma (T_{e}-T_{l}),$$ (4)

where $c_{v,l}$ is the lattice part of the specific heat. For temperatures above the Debye temperature, $c_{v,l}$ is approximately constant and $c_{v,l}\gg c_{v,e}$. Under these conditions, a typical time-dependence of the two temperatures is shown in Fig. 1(b). The low magnitude of the electronic part of the specific heat relative to the lattice part gives rise to sometimes very large laser-induced changes in the electronic temperature (>1000 K) even when the final system temperature increase is relatively modest. Actual experiments are usually performed in a repetitive mode, where it is assumed that the system is in contact with a thermal bath that restores the original temperature by heat diffusion or other transport processes.

Refinements of the basic two-temperature model have been widely applied to describe the changes in the electron energy distribution after femtosecond and picosecond laser heating, particularly with metallic systems where the density of states within about 1 eV of the Fermi surface is large and allows for rapid thermalization of the electrons.^3,4^ Even in systems that are not truly metallic, the basic concepts of the model often allow for a qualitative understanding of transient laser-induced phenomena. As an example of this, we discuss a particular experiment by Möhr-Vorobeva *et al.* where a charge-density wave (CDW) in 1 T-TiSe_2_ that appears below $T_{c}\approx 200$ K was excited by an ultrafast laser pulse in the near-infrared frequency range.^5^ In this material, there is a long-standing debate concerning the origin of the CDW, which largely centers around whether the CDW is driven by some kind of electron-lattice coupling as in a “conventional” CDW or if it is instead a more exotic phenomenon arising from condensation of excitons.^6–12^ In the experiment, x-ray diffraction from the periodic lattice distortion (PLD) created by the CDW was used to detect the amplitude of the CDW as a function of time after the laser excitation. For sufficiently high fluences, the PLD was completely suppressed within 100 fs, indicating that the CDW phase is very rapidly “melted” by electronic excitation. At later times (>10 ps), the diffraction signal partly recovered, but was substantially broader in reciprocal space, suggesting that the CDW reformed, but with a very small correlation length. Furthermore, the energy density needed to transiently melt the CDW in this way was about a factor of 4 lower than would be needed to raise the temperature of the entire system (electrons + lattice) to a value above the transition temperature. The concepts behind the two-temperature model offer an attractive interpretation of these observations under the condensed exciton picture of the CDW. First, the laser heats the electronic subsystem to a very high temperature, sufficient to ionize the excitons or at least to melt the condensate. As the electronic subsystem rapidly cools by transferring heat to the cold lattice, the exciton condensate reforms but with a reduced correlation length. On very long time scales relative to the window of the measurement, the correlation length recovers by a process involving domain wall motion. These results would then appear to be consistent with the exciton condensate view of the CDW phase transition, but leave open the possibility that there could be some contributions from more conventional forms of electron-phonon coupling. Later work using time-resolved THz spectroscopy has indeed suggested that the exciton condensate may be only partially responsible for the PLD.^13^ Qualitatively similar responses can be seen in a variety of materials with CDWs driven by different mechanisms, including those materials where excitons play no known role. For example, a similar picture of CDW regrowth from a melt was recently also applied to understand the growth of the incommensurate CDW from a laser-melted state in 1T-TaS_2_.^14^

**FIG. 2.**
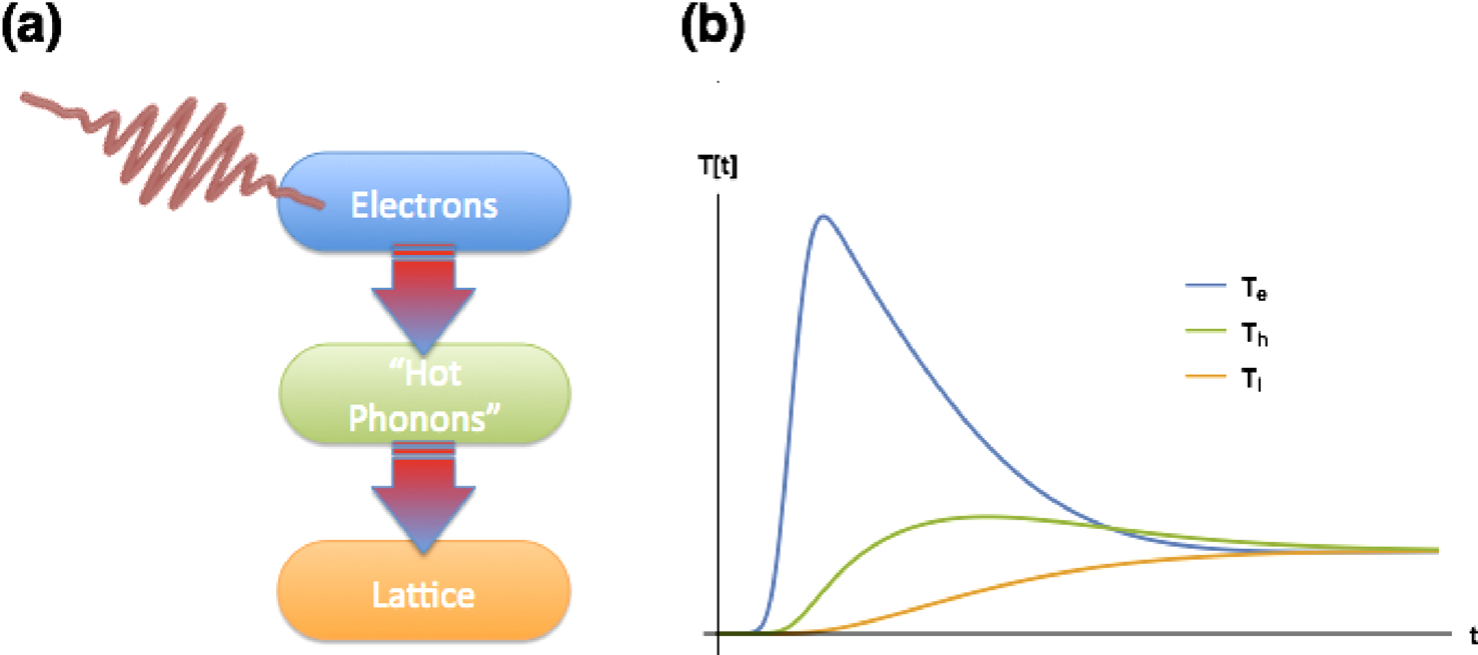
The three-temperature model proposed by Mansart *et al.* to describe electron–phonon interactions in La_2-__*x*_Sr_*x*_CuO_4_. (a) Conceptual sketch of the model. In contrast to the two-temperature model, the electronic subsystem is assumed to interact predominantly with a subset of the phonon modes that form their own subsystem. These “hot phonons” then give heat more slowly to the remainder of the lattice. (b) One possible time-dependence for the electronic, hot phonon, and lattice temperatures *T_e_*, *T_h_*, and *T_l_*. The intermediate hot phonon subsystem delays the heating of the lattice.

Despite the widespread application of the two-temperature model, there are frequently cases where it does not accurately describe the behavior of the electronic or lattice subsystems. This is often addressed by simply expanding the two-temperature model to include additional subsystems. In semiconductors and semimetals, for example, the electrons and holes are sometimes treated as separate subsystems with different temperatures.^15,16^ In materials where the coupling of electrons and phonons is particularly strong for certain phonon modes, it can also be necessary to subdivide the lattice.^17,18^ As an illustrative example with a somewhat unique experimental perspective, we consider a recent experiment by Mansart *et al.*^19^ In this experiment, a femtosecond laser heated the electronic subsystem of the cuprate La_2–__*x*_Sr_*x*_CuO_4_ crystals, for two doping values of *x* = 0.1 and *x* = 0.21. Rather than looking at the transient changes arising from properties of the electronic subsystem, x-ray diffraction from the (400) lattice planes was used to study the response of the lattice by measuring changes in the peak shape and in the Debye-Waller factor, a measure of structural disorder in the crystal that acts to suppress the intensity of diffraction from peaks. Under the assumption that the lattice can be treated at all times as a thermal subsystem, the Debye-Waller factor can be related directly to the lattice temperature. The two-temperature model is, however, not easily applied to this system since the electron-phonon coupling is significantly stronger for a subset of optical phonon modes, resulting in a strongly non-equilibrium lattice state. Following the example of earlier work on modeling time-dependent changes in laser-excited cuprates,^18^ the authors proposed to separate the phonons into two subsystems: one “hot phonon” subsystem containing the optical modes that are strongly coupled to the electrons, and a “lattice” subsystem that contains all other phonon modes including the acoustic modes that are the primary contributors to the Debye-Waller factor changes. The resulting “three-temperature model” is depicted in Fig. 2. Experimentally, these measurements observed a strong dependence of the coupling between the electrons and hot phonons on the excitation density of the laser pulse; this was qualitatively consistent with first-principles calculations, which showed a strong dependence of the coupling on the electronic temperature.

Magnetic systems are another important target for multiple subsystem thermal models. An early application was seen in the interpretation of ultrafast laser-induced demagnetization in various metallic ferromagnets, originally observed in Ni.^20^ but later also seen in a variety of other materials.^21^ The main observation that defines this phenomenon is a rapid, <100 fs decrease of the magnetization in response to heating from an ultrafast laser pulse. Phenomenologically, this was understood in terms of a three-temperature model similar to the one shown in Fig. 2, but with two important differences: (1) instead of the “hot phonon” system, we have a “spin” subsystem that characterizes the magnetism in the material and (2) heat transfer among all three subsystems is considered, although in the implementation of most such models, the coupling of both the electronic and spin subsystems to the lattice is relatively weak. A fast increase in spin temperature drives the demagnetization within this model. Some versions of this also introduce a fourth subsystem by splitting the electronic subsystem into a “hot” and “cold” portion to model the thermal equilibration of the electrons.

**FIG. 3.**
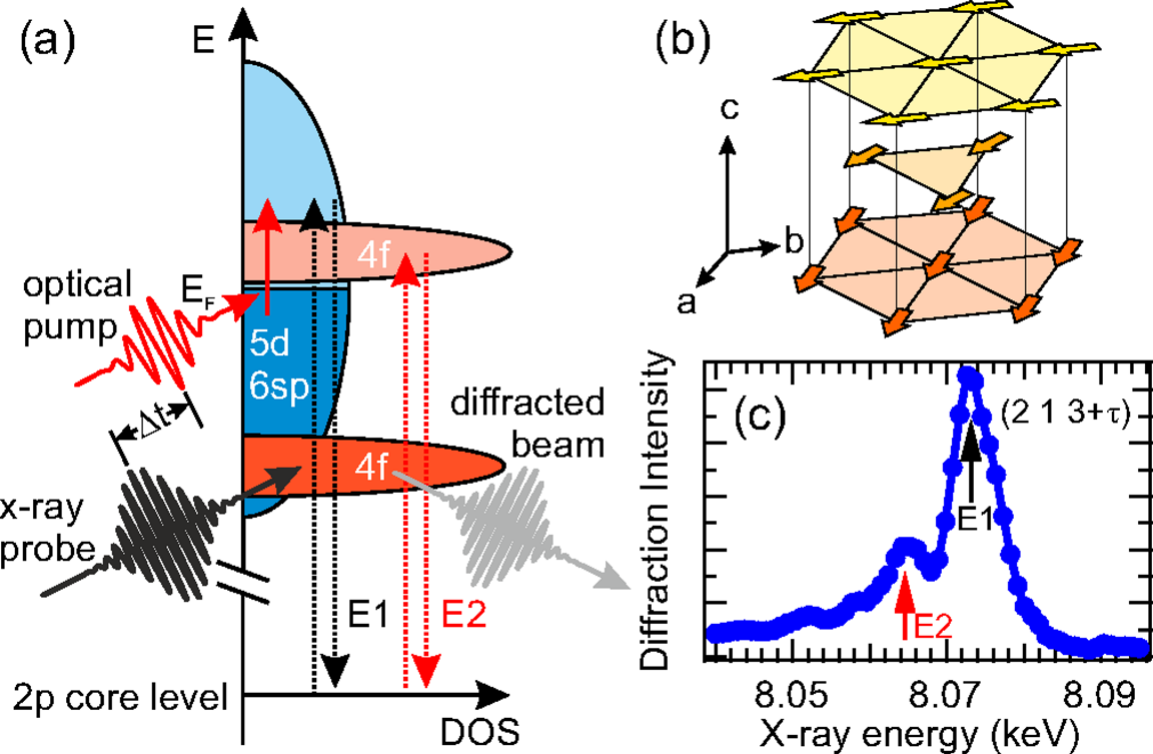
Sensitivity of resonant x-ray diffraction to the transient 5d and 4f magnetic order in elemental Ho after electronic excitation with an ultrashort laser pulse. (a) While the laser pulse excites the 5d electrons directly, resonant x-ray scattering selectively probes 4f or 5d order by tuning the x-ray wavelength to use either 4f or 5d unoccupied states as an intermediate state in the scattering process. The different selection rules for the scattering processes make the 5d intermediate a dipole resonance, whereas the 4f intermediate corresponds to a quadrupole resonance. (b) A sketch of the spin order in Ho in real space, within the hexagonal structural unit cell. The wave vector for the magnetic order is oriented along the crystallographic c axis. (c) Energy dependence of x-ray diffraction at the magnetic ordering wave vector, showing the E1 resonance (5d states) and the E2 resonance (4f states). Reproduced with permission from Rettig *et al.*, Phys. Rev. Lett. **116**, 257202 (2016).^27^ Copyright 2016 American Physical Society.

**FIG. 4.**
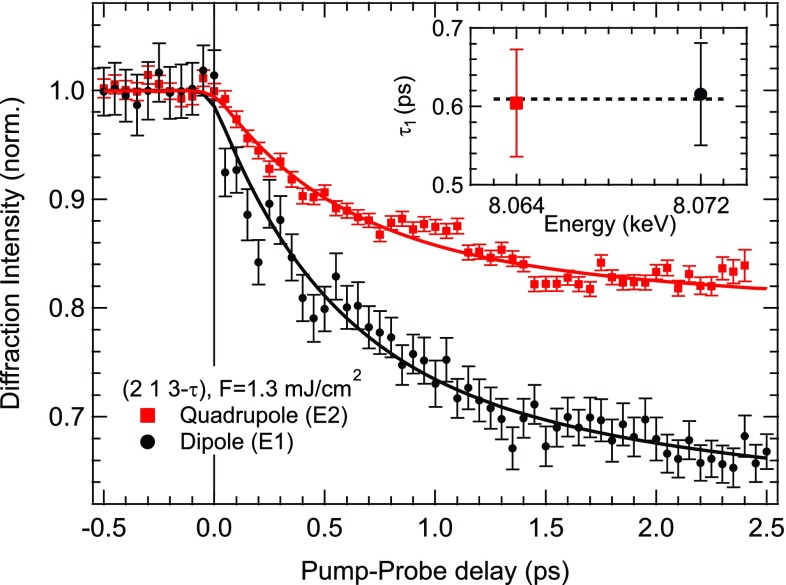
Time-dependence of the intensity of diffraction from the dipole (E1) and quadrupole (E2) resonances of the Ho (2   1   3−τ) magnetic peak. Both decrease, implying a loss of magnetic order for the antiferromangetic sublattice. The curves are fits to a simple exponential decay as an approximation of solutions to a three-temperature model; the inset shows the fit time constants. There is no measurable difference in the sublattice demagnetization of the 4f and 5d spin ordering, suggesting that they are tightly coupled on the time scale of this measurement. The difference in the magnitude for the demagnetization is understood as arising from a difference in the probed volume for the two x-ray energies, which extend to slightly deeper portions of the crystal relative to the 800 nm light used to trigger the sublattice demagnetization. Reproduced with permission from Rettig *et al.*, Phys. Rev. Lett. **116**, 257202 (2016).^27^ Copyright 2016 American Physical Society.

Recently, new methods have made similar studies of magnetic order in antiferromagnetic (AFM) systems possible using resonant x-ray diffraction as a time-resolved measure of the magnetic order. Antiferromagnetic systems have some scientific interest since in these systems there is no net angular momentum in the spins, which may influence the dynamics.^22^ In this context, it is also relevant to take note of the recently studied phenomenon of femtosecond-laser driven switching of magnetism in ferrimagnetic FeGdCo alloys that appears to rely on an ultrafast angular momentum transfer between nanoscale inhomogeneities.^23–25^ Here, a key element of the physics behind this is the interaction of localized f moments and itinerant d electrons which show a different behavior in the transient state.^26^ Recent x-ray diffraction measurements on elemental Ho have further explored this interaction in a system with magnetic order in both the 5d and 4f states but no net magnetic moment.^27^ The magnetic order in Ho at temperatures below $T_{N}\approx 133$ K is characterized by a spin helix structure along the crystallographic c axis with a wave vector $\tau \approx 0.3c^{*}$. X-rays with wavelengths near the L_2,3_ absorption edges are selectively sensitive to this order in either the d or f states (see Fig. 3). The time-dependence of diffraction from each of these resonances at the (2   1   3−τ) magnetic peak is shown in Fig. 4 Diffraction at both resonances decreases with a time scale of about 0.6 ps, with no measurable difference. Under a multi-temperature subsystem model, this implies that the magnetic order for both 4f and 5d electrons are so tightly coupled that they can be identified with a single spin system that characterizes the full magnetic order of the material. This suggests that one essential element of the physics in the switching of f-d ferrimagnets may be elemental heterogeneity, with a spatial separation of f and d electrons.

Similar considerations also apply to laser-driven phase transitions in antiferromagnetic materials. For example, the multiferroic materials TbMnO_3_ and CuO both have at low temperatures multiple antiferromangetic spin-ordered states that arise from frustrated exchange interactions. Excitation of the electronic subsystem in both materials has been shown to drive transitions between different magnetic phases.^28–30^ In TbMnO_3_, Johnson *et al.* performed experiments studying the laser-induced heating of the low-temperature spin-cycloid phase, using resonant x-ray diffraction to monitor the magnetic ordering as a function of time.^30^ For high excitation densities, the magnetic ordering nearly completely disappears on a time scale that decreases with further increasing laser excitation levels. This can be understood in a thermal subsystem model as a transfer of heat from the electronic subsystem to the spin subsystem, forcing the spins to reach temperatures well above the Néel temperature *T_N_*. Although in this model the rate of spin temperature increase is essentially independent of the excitation level from the laser, the spin ordering disappears faster for higher excitations since the spin subsystem reaches *T_N_* at an earlier time. In the case of CuO, a similar kind of spin-cycloid state emerges below $T_{N2}=230\;K$ but is replaced by a different magnetic ordering when cooled further below $T_{N1}=213\;K$. These two magnetically ordered phases can be distinguished by differences in the magnetic ordering wave-vector, which is directly measurable with resonant diffraction at the Cu L edges. Ultrafast laser excitation of the electronic subsystem in the low temperature phase drives a transition between the two antiferromagnetic phases, a process seen in experiments to be limited in time scale to approximately 1/4 of the period of low-wavevector magnetic excitations.^28^ During this process, the electronic temperature reaches several thousand Kelvin, as evidenced by small changes in the shape of the resonance responsible for the magnetic diffraction.^29^

Although thermal subsystem models are a very common way to describe nonequilibrium processes in laser-heated materials, these approaches have some prominent deficiencies that cannot easily be fixed. It is often not clear whether the basic assumption of instantaneous thermal equilibrium within a given subsystem is physically reasonable. For electronic states, this can be partially tested for some systems experimentally using angle-resolved photoemission with femtosecond time resolution.^31,32^ For the lattice, the assumption of a thermalized distribtution of phonon occupation for short times after a sudden heating event is often questionable, but difficult to verify experimentally. Typical phonon lifetimes are on the order of a picosecond, which suggests that true thermalization of the lattice subsystem occurs on significantly longer time scales.

Another issue with “N-temperature” models is their highly phenomenological nature which largely ignores a discussion of the physical mechanisms responsible for redistributing energy among the different excitation channels. This can be important since details of how the interactions take place are sometimes the entire point of a study. Such models are in general quite good for describing time-resolved changes arising from incoherent excitations, but offer limited predictive power since it is often difficult to make a strong connection between any hypothetical mechanisms and the measured coupling parameters.

## III. HYBRID SYSTEMS: COHERENT DYNAMICS DRIVEN BY INCOHERENCE

As just discussed, the thermal subsystem approach sometimes works as a phenomenological description of nonequilibrium changes in a material, but by its nature cannot describe coherent dynamics. Here, by “coherent dynamics,” we mean time-dependent changes in the average value of a structural coordinate that follow an equation of motion, as in classical mechanics. Whereas the thermal subsystem model assumes that different parts of the system are in some kind of quasi-stationary thermal state, coherent dynamics are inherently non-thermal and so cannot be described in such a model.

Sometimes, the time-dependent changes to a material are best understood in a hybrid model that treats some parts of the system statistically and other parts coherently. A common example of this is a situation where an ultrashort laser pulse excites electronic states that rapidly scatter to a subset of other electronically excited states on a time scale shorter than the duration of the pulse. If this population of excited states is coupled to another degree of freedom with a coherence time longer than the duration of the onset of the excitation, this can often be modelled as an effective modulation of the potential energy of this coupled system. In order to describe this situation, we need a model that incorporates both the incoherent and coherent elements of the time-dependent response. One way to accomplish this is to still treat portions of the material as a thermodynamic subsystem, but to model the coupling of that subsystem to other degrees of freedom as a sudden change in the effective potential energy landscape that stimulated coherent dynamics: essentially a “hybrid” model combining time evolution of statistical populations with coherent dynamics. This approach is best explained through examples.

**FIG. 5.**
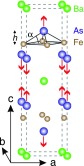
Sketch of the unit cell of BaFe_2_As_2_, showing both the eigenvector (atomic displacement pattern) of the A_1__*g*_ mode discussed in the text as well as its relationship to the angle *α* of the As-Fe bond to the Fe atom planes. Reproduced with permission from Rettig *et al.*, Phys. Rev. Lett. **114**, 067402 (2015).^33^ Copyright 2016 American Physical Society.

**FIG. 6.**
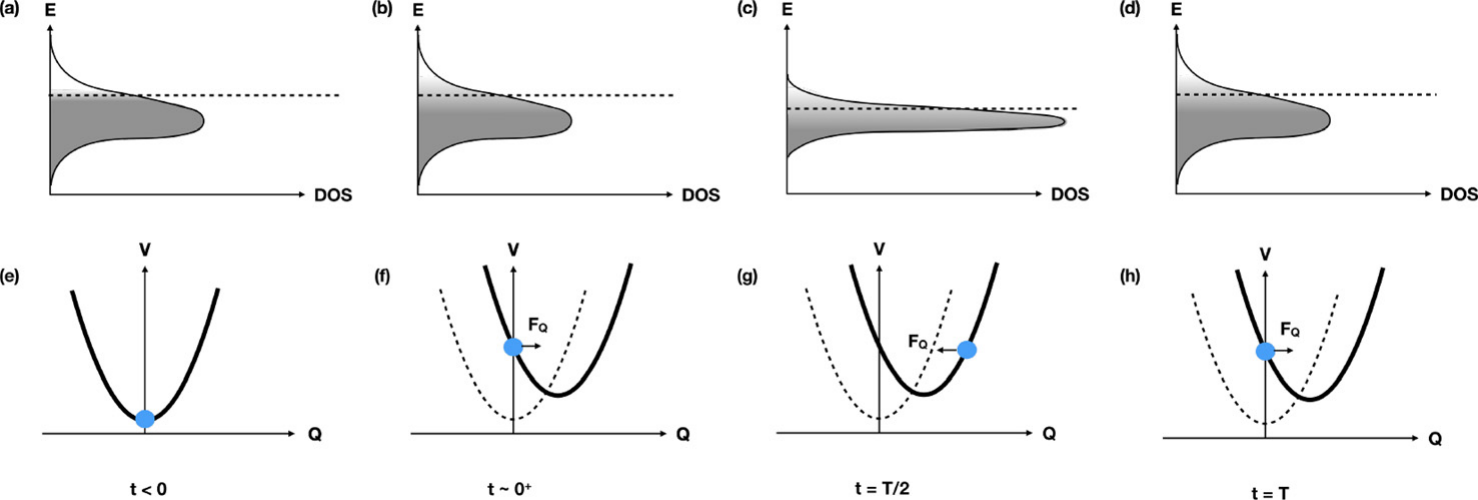
Conceptual sketch of one mechanism of DECP, in rough correspondence to recent observations in BaFe_2_As_2_.^33,34^ Panels (a)–(d) show the density of electronic states (DOS) at various points in time relative to a pump laser that suddenly raises the electronic temperature at *t* = 0. The dashed line shows the energy of the chemical potential *μ*, and the greyscale indicates the occupation of the electronic states. Panels (e) and (f) show the corresponding potential energy versus a relevant phonon coordinate. At times *t* < 0, the temperature of the electrons is low and the band is mostly full; the phonon mode coordinate sits at its equilibrium position. Just after excitation [panels (b) and (f)], the electronic temperature increases causing some higher energy states to increase occupation and some lower energy states to become vacant. Deformation potential coupling of these electronic states to the phonon mode causes a shift in the minimum of the phonon mode potential energy, which in turn leads in a classical picture to a force that drives the phonon mode coordinate away from its initial value. After half of a vibrational period *T*, the distortion of the crystal structure from the phonon mode coordinate change causes a narrowing of the electronic bandwidth, which shifts the position of the chemical potential. After one full phonon period, the original atomic and electronic structure is recovered, beginning the cycle again. The changes to the bandwidth and chemical potential are greatly exaggerated for illustrative purposes.

One broad class of hybrid models concerns cases where a laser pulse has excited electronic states with a very short coherence time that nonetheless couple to coherent vibrational modes. This is well illustrated by a pair of recent experiments on single crystals of BaFe_2_As_2_, a material known as a parent compound for Fe-pnictide superconductors.^33,34^ In both experiments, a femosecond laser pulse at 800 nm center wavelength excites electronic states in this poorly conducting metallic material. The experiments differ in how they probe the resulting time-dependent changes. Yang *et al.*^34^ use femtosecond angle-resolved photoemission (ARPES) to track the energy and occupation of electronic states. For an absorbed laser fluence of 0.47 mJ/cm^2^, the measured photoemission spectrum shows evidence of a strong 400 K effective temperature increase of the electronic states on a time scale of <100 fs. This temperature relaxes on a time scale of several hundred femtoseconds, quite consistent with what one might expect from a simple two-temperature model. On top of this electronic temperature change, however, another time-dependent change is observed: a prominent spectral feature that Yang *et al.* use to identify the position of the chemical potential *μ* oscillates with a periodicity of about 185 fs, matching the periodicity of an A_1__*g*_ symmetry optical phonon mode that involves a motion of the As ions along the c-axis of the crystal (see Fig. 5). This vibrational mode of the bulk crystal is excited coherently by the fast increase in the electronic temperature. Essentially, the redistribution of electronic state occupation among the bands near the Fermi energy alters the interatomic force balance that defines the equilibrium crystal structure. In the electronically “hot” state, the positions of the As ions have an effective potential energy minimum at a location displaced slightly from their average positions in the low-temperature equilibrium state. This causes the As atoms to oscillate around the position of their new potential energy minimum, just like a classical harmonic oscillator would oscillate coherently in response to a displacement from its equilibrium position. As the As atoms oscillate coherently about their new position, the electronic bands near the Fermi surface which are coupled to this vibrational mode also distort and cause a coherent, time-dependent shift in the ARPES spectral features. In this particular case, Yang *et al.* make the somewhat unusual claim that this time-dependent change is direct evidence of an oscillation in the chemical potential itself. Figure 6 shows a simplified sketch of the entire process under this interpretation, which is a particular case of a more general phenomenon known as displacive excitation of coherent phonons (DECP). Similar DECP-related phenomena have been observed in time-resolved ARPES experiments on other materials,^36–39^ but in these cases, the time-dependent changes are interpreted as a consequence of periodic modulation of the band structure and not of the chemical potential.

**FIG. 7.**
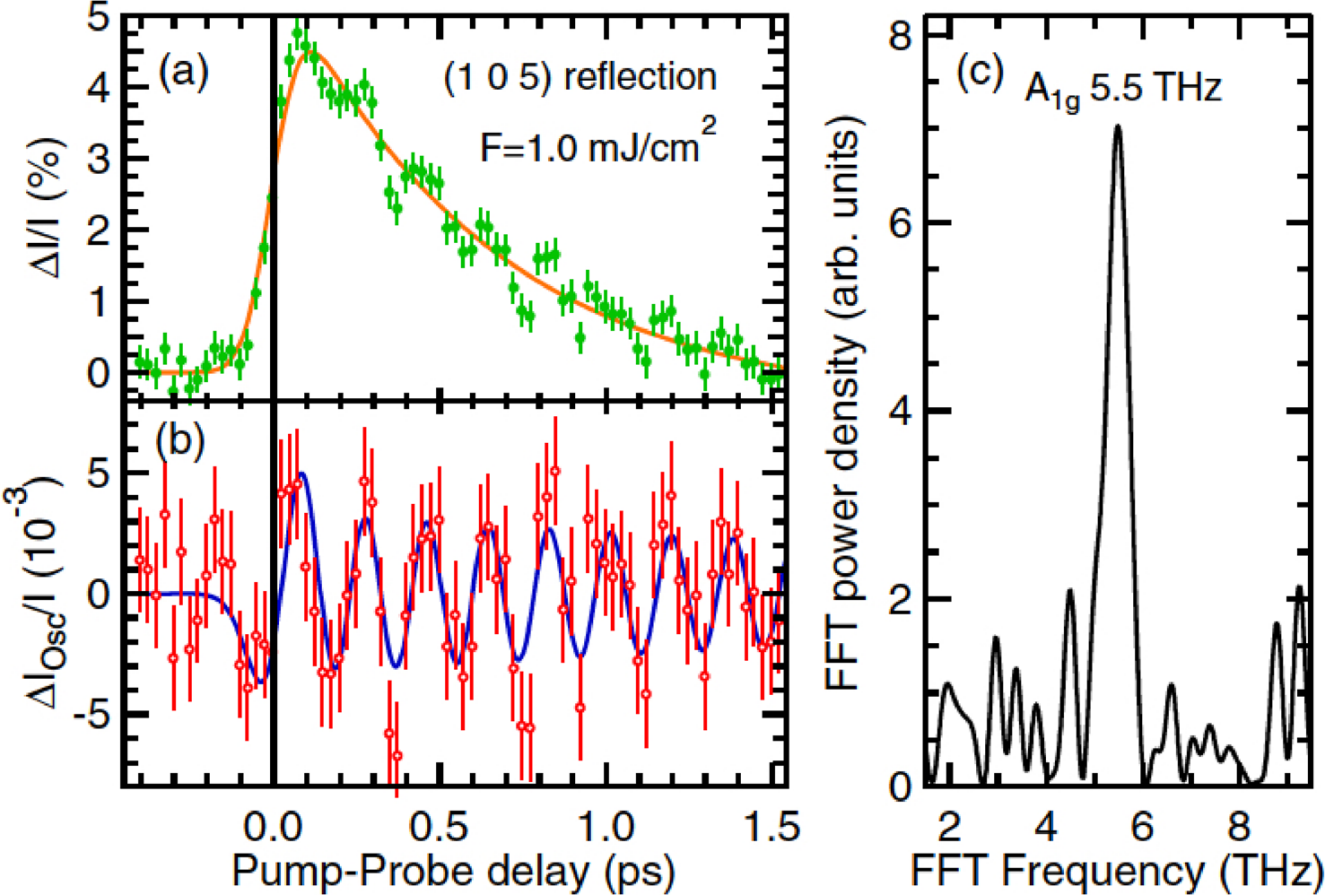
Time-dependent changes in the x-ray diffracted intensity from the (1 0 5) planes of BaFe_2_As_2_. (a) Change in intensity versus time for an excitation fluence of 1.0 mJ/cm^2^. The solid curve indicates the estimated non-oscillatory background arising from the displacement of the mode coordinate. (b) Oscillatory component of the changes, showing also a fit to a model of a damped simple harmonic oscillator. (c) Fourier transform of the data in (b), showing a strong peak at the 5.5 THz frequency of the A_1__*g*_ mode. Reproduced with permission from Rettig *et al.*, Phys. Rev. Lett. **114**, 067402 (2015).^33^ Copyright 2016 American Physical Society.

In addition to the time-dependent shift of *μ*, the corresponding atomic motion can be seen by x-ray diffraction.^33^ Figure 7 shows a sample of the data, where measurements of the intensity of both the (1 0 5) and (2 0 6) reflections show time-dependent changes in the structure factor due to the A_1__*g*_ mode. For the (1 0 5) peak, the changes are characterized by an increase in diffracted intensity and an overall oscillation about a new average, whereas for the (2 0 6) reflection, the changes are similar but show an overall decrease with oscillations that are out of phase with respect to the (1 0 5) peak. This difference in response is consistent with the changes that distortion of the crystal structure along the A_1__*g*_ coordinate has on the scattering efficiency of these diffraction peaks. These data coupled with the ARPES data give a direct experimental measurement of the electron-phonon coupling in this material. In this particular case, understanding the magnitude of this coupling is highly relevant since this particular A_1__*g*_ mode modulates the angle *α* between the Fe-As bond and the Fe planes, which in turn controls the magnetic moment of the Fe ions. Coherent control of the Fe magnetic moments using this phonon mode has been suggested as an explanation for previous observations of a transiently generated spin density wave phase created by optical excitation.^40^

**FIG. 8.**
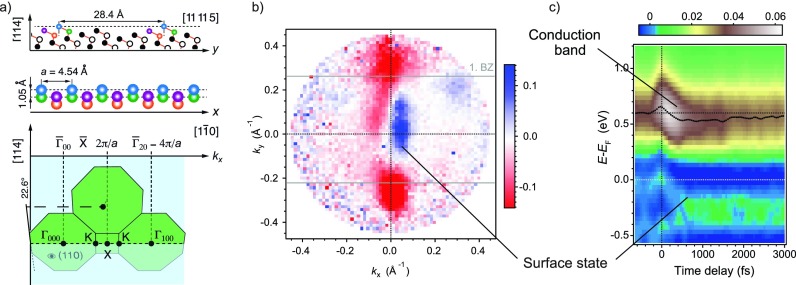
Atomic and electronic structure of Bi(114). (a) Real space (top) and reciprocal space lattice (bottom) along the atomic chains. (b) Fermi surface taken with circularly polarized 6 eV photons: The atomic chains support an electronic surface state which is spin-split as shown by polarization dependent measurements. The two vertical lines are colored owing to the sign of the asymmetry measured by circular dichroism in photoemission. (c) Time-resolved photoemission spectra after normalization by the Fermi-Dirac distribution. Panels (a) and (c) adapted with permission from Leuenberger *et al.*, Phys. Rev. Lett. **110**, 136806 (2013).^41^ Copyright 2013 American Physical Society.

Sudden changes in the electron temperature can drive not only coherent vibrations in bulk modes, but also coherent surface modes. This was recently shown in ARPES measurements of the (114) surface of bismuth single crystals.^41^ The (114) surface of Bi is a peculiar example of a nanoscale surface reconstruction: cut at 56° from the low-index (111) surface, it exhibits monatomic rows with a lattice constant of 4.5 Å separated by large terraces of about 28 Å. As a consequence, the surf ace state has almost perfect one-dimensional (1D) dispersion with parallel Fermi lines of opposite spin polarization,^42^ one per Brillouin zone. The metallic surface electronic structure thus strongly resembles the surface states of topological insulators, as shown in Fig. 8.

**FIG. 9.**
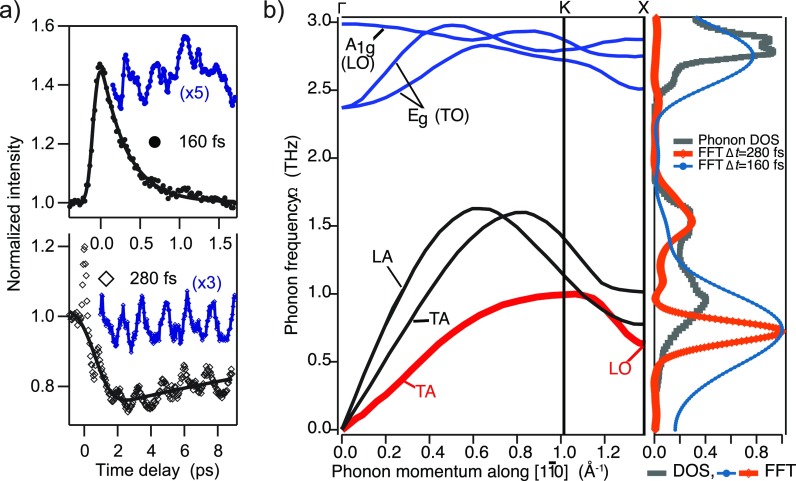
Coherent phonon modes in Bi(114). (a) Observation of coherent phonon oscillations in photoemission intensities from bulk (top panel, pump pulse duration 160 fs) and surface states (bottom, pump pulse duration 280 fs). The blue data points are the modulations obtained by removing a smooth function (black lines) from the raw data (black symbols). Adapted with permission from Leuenberger *et al.*, Phys. Rev. Lett. **110**, 136806 (2013).^41^ Copyright 2013 American Physical Society. (b) Phonon dispersion curves along ΓKX from Ref. 16. In the right panel, the phonon DOS is shown together with the Fourier transforms for both different pump pulse durations (orange open symbols and thick line 280 fs, small dark blue solid symbols 160 fs).

Figures 9(a) and 9(b) show the time dependence of the photoemission signal from both bulk and surface electronic states in this material. In most cases, time-resolved photoelectron spectroscopy has shown that only bulk states are modulated in photoemission spectra. Surface states are largely decoupled from the bulk and only experience shifts and bleaching to the electronic excitations.^43^ The situation is different in Bi(114) for lattice modulations along the chains: here, the nearly 1D dispersion together with strong coupling renders the atomic chains unstable against dimerization of neighboring atoms as seen in low-temperature scanning tunnelling microscopy (STM) images.^35^ As a consequence, femtosecond laser-induced heating of the electronic states causes a modulation of the surface state photoemission signal by a low-frequency phonon at about 0.7 THz. By temporal broadening of the infrared pump pulse to a width larger than the half cycle of the bulk A_1__*g*_ phonon (with a frequency of about 2.8 THz), it could be shown that the 0.7 THz mode was directly excited by the pump pulse via displacement of electronic charge rather than by decay of high-energy optical modes.^41^ A comparison with calculated phonon dispersion curves^16^ along the direction of the atomic rows, ΓKX, reveals that the dispersion becomes flat at the midpoint X between two adjacent reciprocal lattice points. The character of these branches is optical with both transverse and longitudinal modes in close proximity. The excitation of a coherent mode here corresponds to a standing wave obtained by superposition of LO modes at the X-points with opposite wave vectors. The dimerization of atoms observed at low temperature may be the result of an interaction of the electronic system with this mode. In the case of full phonon softening, this would lead to a periodic lattice distortion as in other charge-density wave systems.^44^ The fact that no long-range order or phase transition is observed in low-temperature scaning-tunneling microscopy (STM) may be rationalized by the opposite spin polarization of the parallel sheets of the Fermi surface which inhibit efficient nesting of the Fermi surface.^41^

In certain systems, laser-induced electronic excitation of incoherent, quasi-thermal populations can lead to a coherent structural motion that transiently alters the symmetry of the material. A relatively simple example of such dynamics was recently observed in K_0.3_MoO_3_, otherwise known as “blue bronze.” Blue bronze is a quasi one-dimensional metal at room temperature. When cooled to temperatures below *T_c_* = 183 K, the material becomes unstable to a small incommensurate distortion of the lattice that creates a gap in the electronic DOS at the Fermi energy, resulting in the formation of a CDW.^45,46^ Unlike the CDW in TaSe_2_, the physical origin of the lattice distortion in blue bronze is widely recognized as the result of a Peierls instability arising from the large parallel regions of the Fermi surface that are a natural consequence of nearly one-dimensional conductivity in the high-temperature phase.

**FIG. 10.**
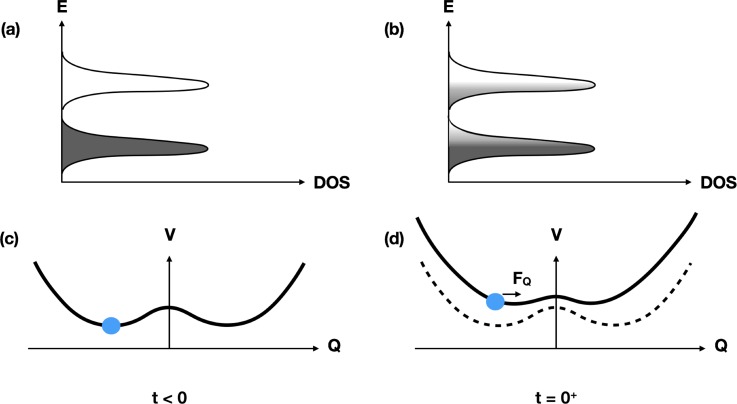
Conceptual sketch of the behavior of a Peierls-distorted system upon weak electronic excitation. Panels (a) and (b) show the density of electronic states for the gapped system, before and just after electronic excitation, respectively. After excitation, there are significantly more electrons in the conduction band and holes in the valence band. Panels (c) and (d) show the influence this has on the interatomic potential plotted as a function of the magnitude of the incommensurate lattice distortion. Before excitation, the lattice distortion has two equivalent minima that correspond to out-of-phase distortions of the lattice. Here, setting *Q* = 0 gives the undistorted structure, as would appear at high temperatures. The rearrangement of electronic states from the excitation process results in the modified potential energy surface shown in (d) which still has two equivalent minima but shifted slightly closer to *Q* = 0. If the electronic excitation is fast compared with the vibrational period associate with this coordinate, the coordinate will oscillate around the new local minimum.

Figure 10 shows on a conceptual level what happens when this system is weakly excited with a laser pulse. Initially, the electronic structure is that of a semiconductor: there is a gap in the electronic density of states, and at low temperatures, only very few electrons and holes are present. Figure 10(c) shows the corresponding interatomic potential energy as a function of the vibrational coordinate that describes the incommensurate modulation of the lattice; in this cold state, there are two equivalent minima in the potential energy surface, corresponding to equal magnitudes of lattice distortion but with opposite phases. In equilibrium, this vibrational coordinate is very close to one of these minima, resulting in a charge density modulation. After electronic excitation from the laser, there are suddenly more electrons in the conduction band and more holes in the valence band. Since these states are strongly coupled to the CDW, the potential energy surface with respect to the lattice distortion coordinate changes, resulting in a shift of the minima to a position closer to the undistorted value (at *Q* = 0), as shown in Fig. 10(d). Assuming that the electronic state relaxation to the low temperature state is slow, the structure will start to oscillate around the new minimum. This is essentially the same result that we saw for BaFe_2_As_2_.

**FIG. 11.**
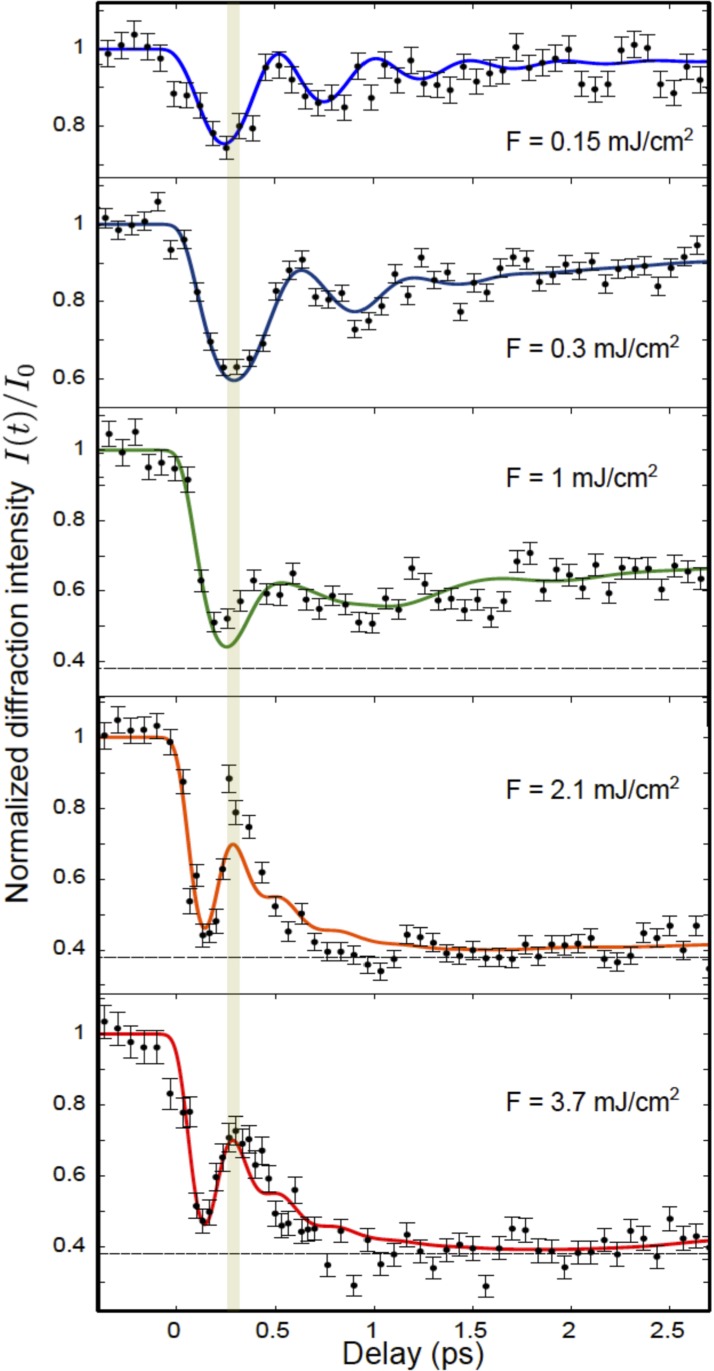
Dependence of the time-resolved diffraction from the superlattice of K_0.3_MoO_3_ on pump laser fluence. The vertical bar indicates the time of the first minimum for the fluence of 0.3 mJ/cm^2^. The solid curves are a fit to a model based on the discussion in the text. Adapted with permission from Huber *et al.*, Phys. Rev. Lett. **113**, 026401 (2014).^47^ Copyright 2014 American Physical Society.

As with our previous examples, x-ray diffraction can track the coherent dynamics of the lattice distortion. The periodic lattice distortion in blue bronze gives rise to several additional x-ray diffraction peaks that are not present in the high temperature phase. The intensity of these peaks is proportional to the square of the amplitude of the modulation, providing a very clear, quantitative measure of the lattice distortion. Figure 11 shows the intensity of time-resolved x-ray diffraction from the (1 3.252 −0.5) superlattice peak^47^ under different levels of laser excitation. For low values of the excitation, we see oscillations around a reduced intensity value, just as we would expect from the process sketched in Fig. 10. At high values of the laser excitation, however, the dynamics start to look quite different. At the highest excitation fluence, the intensity drops quickly, then rapidly recovers, and then drops again to a somewhat constant level. The timing of the temporary recovery in intensity coincides with the first minimum of the coherent oscillations at low excitation levels.

**FIG. 12.**
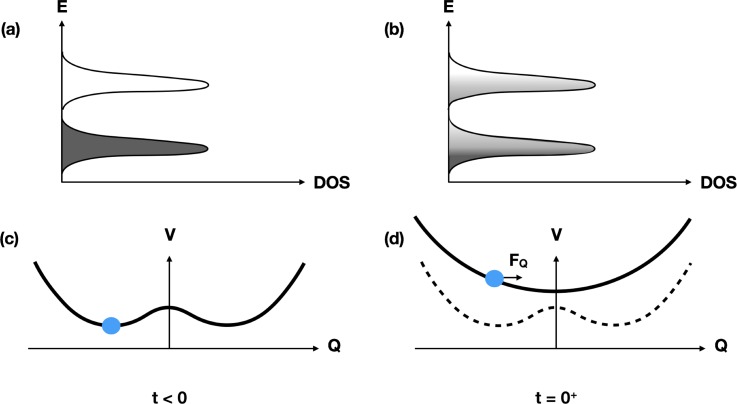
Conceptual sketch of the behavior of a Peierls-distorted system under strong electronic excitation, as Fig. 10 shows for the case of weak excitation. As in that figure, panels (a) and (b) show the electronic DOS and occulation levels before and just after excitation, while panels (c) and (d) show the corresponding potential energy surface for the amplitude of the lattice distortion. In this case, the excitation is so strong that the gap becomes unstable, redefining the shape of the potential energy surface such that the undistorted, *Q* = 0 structure is now the minimum. Assuming that the electronic excitation is fast compared with the period of vibrations in this potential, the modulation coordinate *Q* will accelerate toward and through zero, causing a 180° reversal of the phase of the lattice distortion. This explains the fast transient dip and recovery seen in the data of Fig. 11.

Figure 12 shows another conceptual sketch of what happens at high excitation levels that can explain this behavior. The basic difference compared with the situation at low excitation levels shown in Fig. 10 is that the large number of excited electron-hole pairs makes the undistorted crystal more energetically favorable, replacing the double-well potential seen at low temperatures with a single-well-potential. The structural distortion coordinate begins to oscillate around this undistorted position with essentially the same period *T* as before, but since x-ray diffraction is sensitive only to the square of the coordinate, there is a minimum in the diffracted intensity at t=T/4 rather than at t=T/2. After passing through this minimum, the distortion “overshoots” to a distortion with the opposite phase, and then finally returns to the undistorted phase where it stops moving due to the onset of a strong damping from coupling to other excitations that are enhanced by a drop in the electronic temperature to a value near *T_c_*. The result after about 1 ps is a transient higher symmetry structure, similar to what is obtained by slowly heating the system adiabatically across the phase transition temperature.

**FIG. 13.**
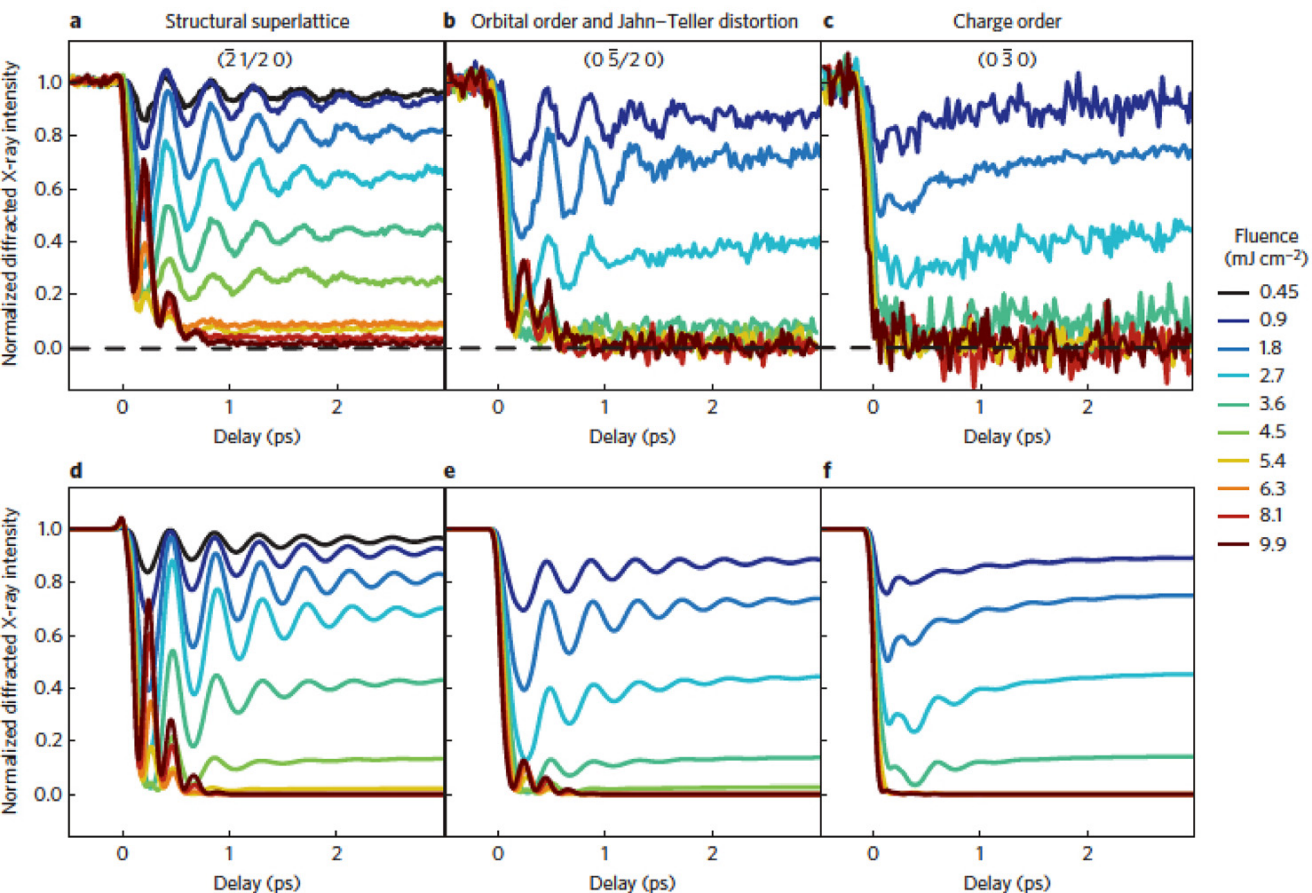
Time-dependent changes in the valence and superlattice order in Pr_0.5_Ca_0.5_MnO_3_ after electronic excitation with varying laser fluence. Panels (a)–(c) show the experimentally measured normalized diffracted intensity from reflections that are sensitive to the structural superlattice, the orbital order and Jahn-Teller distortion, and the charge order, respectively. Panels (d)–(f) show the results of a model calculation where the charge order is considered to drive the other orderings in the crystal. Reproduced with permission from Beaud *et al.*, Nat. Mater. **13**, 923 (2014).^49^

A somewhat more complicated example of an ultrafast, electronically driven change in symmetry is offered by the mixed-valence manganites.^48,49^ We will specifically discuss the case of Pr_0.5_Ca_0.5_MnO_3_, which at high temperatures has an orthorhombic crystal structure that can be seen as a small distortion of a cubic perovskite. Cooling this material below $T_{COO}\approx 220$ K causes the valence charge and orbitals of the Mn ions to order spatially, causing a small distortion of the lattice from Jahn-Teller coupling that makes the crystal symmetry monoclinic with a doubling of the unit cell along its b-axis. Since this transition is highly sensitive to the Mn ion valence electrons, one would expect this material to be a good candidate for seeing large scale structural changes triggered by excitation of electronic states. Indeed, Fig. 13 shows the results of an x-ray diffraction measurement where different peaks that are sensitive to different components of the low temperature ordering are measured near the Mn K-edge resonance, which enhances sensitivity to orbital, charge and Jahn-Teller orderings. As with the blue bronze experiment, low excitation levels cause long-lived oscillations in the structural components, whereas at high excitations, the frequency of oscillations doubles and also damps out faster. In this case, the signal-to-noise level is sufficient to see several high frequency oscillations in the high-symmetry phase. One uniquely interesting aspect of this measurement is the ability to measure the charge order directly. The data show that the charge order drops almost instantly, consistent with the expectation that the pump pulse excites the Mn d-states and directly melts charge ordering in the material. The lower panels of Fig. 13 show a model based on the idea that the charge order serves as a primary order parameter for the transition, which then couples dynamically to the structural components of the low-temperature distortion. The agreement with the data is quite striking, suggesting that this is a valid approach to understand complex coupled phase transitions involving multiple measurable order parameters.

As noted in Sec. II, magnetically ordered materials can also undergo phase transitions induced by laser heating. In some cases, these also contain a component of structural coherence that may be bound to the magnetism.^50,51^ A prime example of this is the phase transition between the low-temperature antiferromagnetic (AFM) and the high temperature ferromagnetic (FM) phase in FeRh, a crystal with a cubic CsCl structure. In this material, the equilibrium magnetic phase transition from AFM to FM is first order and accompanied by an isotropic expansion of the unit cell volume by approximately 0.5%.^52^ A long-standing question concerns the mechanism of the phase transition, specifically regarding the connection between the large lattice dilation and the magnetic ordering. Recently, time-resolved methods have been applied to study this connection.^50,53–55^ Relevant to our discussion above, Mariager *et al.* used both time-resolved x-ray diffraction and Kerr rotation measurements to track both the structural changes and the time-dependence of the average magnetic moment induced by laser heating thin films of FeRh just below the equilibrium phase transition temperature.^50^ The structural measurements show a shift of the lattice constant from both a prompt expansive stress due to heating and a nucleation and growth process for the new phase. The fast stress leads to small coherent acoustic oscillations that are visible in the x-ray diffraction and indicate the presence of a coherent strain wave that moves from the surface into the bulk at the speed of sound. The lattice expansion is complete within 100 ps. Accompanying measurements of the magnetic dynamics, however, show a much slower increase over a time scale of 200–300 ps. This was interpreted as the time scale needed for small FM domains with initially random orientation to align in the applied field.

## IV. FULLY COHERENT DYNAMICS

Rather than acting through excited electronic states, in some cases it is possible to use electromagnetic radiation to more directly drive coherent motion. One of the simplest examples is using carrier-envelope phase stable mid- and far-infrared frequencies to drive vibrational modes that couple in first order to an oscillating electric field: in other words, infrared active phonon modes. In this case, the electromagnetic field imprints its coherence onto the lattice, behaving just as a driven harmonic oscillator. The resulting coherent motion can be even seen using time-resolved x-ray diffraction; a recent example is given by Kozina *et al.* who show the actual atomic motion associated with THz-field driven dynamics of a vibrational mode in SrTiO_3_.^56^

**FIG. 14.**
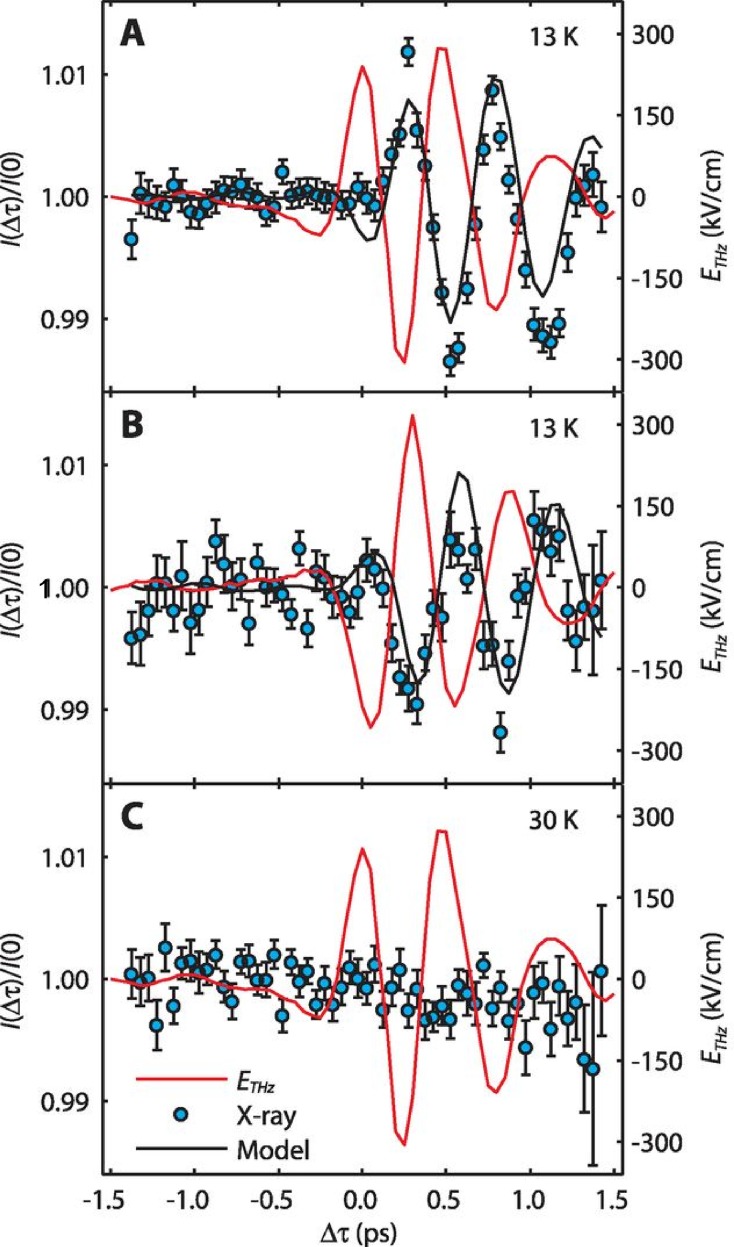
The time-dependent response of magnetic diffraction from the incommensurate magnetic order in TbMnO_3_ during coherent excitation of an electromagnon near 2 THz. In all panels, the data points indicate the diffracted intensity, and the red solid curves show the electric field incident on the surface of the crystal. Panels (a) and (b) show the low-temperature behavior, where the excitation of the electromagnon drives a coherent change in the magnetic structure with the same frequency as the driving field. Panel (c) shows what happens when the temperature is increased to 30 K, where the materials are no longer multiferroic and the electromagnon is suppressed; here the magnetic response is also strongly suppressed. Adapted with permission from Kubacka *et al.*, Science **343**, 1333 (2014).^57^

Another more involved example of a THz-driven excitation was reported by Kubacka *et al.* for electromagnon excitations in multiferroic TbMnO_3_,^57^ the same material where we discussed electronic state-induced phase transtions in Sec. II. Electromagnons are electric-dipole active excitations of spins, made so by strong magneto-electric coupling. It has theoretically been predicted that a strong high-field single cycle THz pulse with several tens of MV/cm field strength at resonance frequency of an electromagnon leads to a canting of the spin cycloid which is followed by a reversal of the cycloid rotation direction within a few ps.^58^ Due to the magnetoelectric coupling, the polarization is expected to reverse on similar timescales. Reference 57 explores the possibility of such switching using resonant soft x-ray diffraction to measure the motion of the spins when this electromagnon is driven by an intense THz field. Figure 14 shows the main result, showing coherent spin dynamics in the low temperature multiferroic phase where the electromagnon exists, at temperatures below 28 K. An analysis of the results indicates that the THz field is able to drive a ±4° rotation of the spin cycloid, which is roughly consistent with the predictions of Ref. 58.

It is also possible to use coherently driven modes to drive other changes in a material. When the mode driven directly by the electromagnetic field is a vibrational mode, this is termed “nonlinear phononics,” since the coupling of this driven mode to other excitations is necessarily the result of a nonlinear coupling term in the resulting equations of motion.^59,60^ The general concepts have been recently reviewed in Ref. 61. In some respects, nonlinear phononics behaves similarly to electronically driven transitions, particularly in the case where the driven mode has a much higher frequency than the mode it couples to. In this case, strong excitation of the high frequency mode drives a displacive excitation of the low-frequency mode, just as we saw for the examples given in Sec. III. The coherent lattice motion induced by this process has been observed using time-resolved x-ray diffraction in a model system.^62^ Relative to electronic excitation, there is, however, a strong advantage to using phonon nonlinearities to drive structural modifications. Since vibrations generally involve low-energy modes that have long coherence times, this type of excitation can deliver far less overall heating to the material while still producing the desired effects. This is important if the primary interest is in driving phase transitions to states that are electronically delicate. In fact, there has been considerable recent work focusing on using nonlinear phononics to drive different materials into states that have the low-frequency spectroscopic properties of superconductors.^63–66^ For this type of control, strong electronic excitation is undesirable since the generation of hot quasiparticles would easily destroy any superconducting condensate.

## V. SUMMARY AND OUTLOOK

In Secs. II–IV, we have given several examples of how momentum-resolved probes using x-rays and photoelectrons can give quite detailed and specific information on time-resolved changes in solid state materials. In situations where the time-dependent changes are largely incoherent and typically only understandable using a statistical approach, ARPES is well-suited to characterizing the transient electronic temperature. At the same time, x-ray diffraction measurements at diffraction peaks are able to quantitatively measure contributions of disorder in the lattice or, in the case of antiferromagnets, the spins. Coherent excitations that are either triggered by these incoherent statistical changes or perhaps driven directly by an electromagnetic field can also be observed. X-ray diffraction based measurements are directly related to changes in the long range order and so provide a very quantitative view of coherent changes in structure or magnetism. ARPES can also see evidence of these coherent excitations by their influence on the electronic states. Although we have not discussed it in this review, photoelectron diffraction is another recently developed technique that also has the capability to see coherent structural changes in short-range structural order more directly.^67,68^

Looking forward, there are several promising directions for future development. One is the improvement and increase in accessibility of high intensity short pulse x-ray sources that make many of the experiments related here possible. Improvements in time-resolution down to 10 fs or less are particularly fruitful, giving the possibility to observe coherent effects in a much wider variety of modes. Most of the experiments reported here had an effective time-resolution of around 100 fs, which limits the range of accessible coherent vibrational modes to the low-frequency end of the spectrum. Another area of improvement is the development of new techniques to look more selectively at different excitation populations, both coherent and incoherent. A variety of new techniques promise to extend inelastic x-ray scattering into the time domain, allowing the measurement of the transient momentum dispersion relations of excitations.^69,70^ This would be a qualitative improvement on the work reported in this review, where the experiments that characterize structural or magnetic dynamics were limited to looking at the effects of the dynamics on the long range order. A more complete view of excitations at high spatial frequencies could offer better insights into whether, for example, a particular subsystem is really in thermal equilibrium or whether it should instead be considered as a strongly out of equilibrium part of the material. One additional frontier in this area is connected to the development of stronger low-frequency electromagnetic pulses that may be able to drive nonlinear dynamics outside a weakly perturbative regime. There is already some promising work in this area on driving dynamics in ferroelectrics,^56,71^ but there is likely much more to be done.
